# Comparative Efficacy and Safety of Saroglitazar and Dapagliflozin on Hepatic Steatosis and Glycemic Control in Patients With Metabolic Dysfunction‐Associated Steatotic Liver Disease: A 25‐Week Prospective Multicenter Comparative Cohort Study

**DOI:** 10.1002/edm2.70377

**Published:** 2026-11-01

**Authors:** Mukulesh Gupta, Kumar Prafull Chandra, Rajiv Awasthi, Arunkumar R. Pande, Nitin Ranjan Gupta, Vivek Agarwal, Dinesh Kumar

**Affiliations:** ^1^ Udyan Health Care Pvt Ltd Lucknow Uttar Pradesh India; ^2^ Dr Chandra's Diabetes and Heart Clinic Lucknow Uttar Pradesh India; ^3^ Prarthana Clinic and Diabetes Care Centre Lucknow Uttar Pradesh India; ^4^ Lucknow Endocrine Diabetes & Thyroid Clinic Lucknow Uttar Pradesh India; ^5^ Lucknow Hormone Centre Lucknow Uttar Pradesh India; ^6^ RR Diabetes and Heart Care Centre Lucknow Uttar Pradesh India; ^7^ Harsha Clinic and Diabetes Centre Lucknow Uttar Pradesh India

**Keywords:** dapagliflozin, MASLD, saroglitazar

## Abstract

**Introduction:**

Metabolic dysfunction‐associated steatotic liver disease (MASLD) is the leading cause of chronic liver disease globally, driven by rising obesity and type 2 diabetes mellitus (T2DM). Existing medications provide minimal fibrosis regression, highlighting the need for comparative evidence on agents with metabolic and hepatic benefits. This study compared the efficacy and safety of saroglitazar 4 mg and dapagliflozin 10 mg in patients with MASLD.

**Methods:**

This 25‐week, prospective, multicentre, open‐label active‐controlled comparative cohort study enrolled adults aged 18–70 years with MASLD confirmed by FibroScan (steatosis grade S3, controlled attenuation parameter [CAP] ≥ 292 dB/m, and/or fibrosis stage ≥F2, liver stiffness measurement [LSM] ≥ 8.0 kPa). Participants received saroglitazar (*n* = 27) or dapagliflozin (*n* = 27) once daily, added to standard of care per physician discretion. The primary endpoint was change from baseline in hepatic steatosis (CAP) and fibrosis (LSM); secondary endpoints included HbA1c, hepatic enzymes, lipid profile, and adverse events.

**Results:**

All 54 participants completed follow‐up. CAP decreased from 314.7 to 278.7 dB/m with saroglitazar and from 310.2 to 287.0 dB/m with dapagliflozin (between‐group *p* = 0.44). LSM decreased in both arms (saroglitazar −0.96 kPa; dapagliflozin −0.46 kPa; between‐group *p* = 0.80). HbA1c improved in both groups (saroglitazar 8.28%–6.76%; dapagliflozin 8.14%–7.30%), with a significantly greater reduction with saroglitazar (between‐group *p* = 0.04). ALT and AST declined similarly in both arms despite higher baseline ALT with saroglitazar. Triglycerides decreased significantly within both groups (saroglitazar −62.5 mg/dL; dapagliflozin −28.3 mg/dL), with no significant between‐group difference. No treatment‐emergent adverse events, including edema, occurred in either group.

**Conclusions:**

Both saroglitazar and dapagliflozin improved hepatic steatosis, fibrosis markers, liver enzymes, and glycemic control over 25 weeks, with comparable hepatic benefit. Saroglitazar produced a significantly greater reduction in HbA1c, suggesting a possible glycemic‐preferential advantage in patients requiring simultaneous glycemic and hepatic control; larger randomized trials are needed to confirm any differential hepatic efficacy.

**Trial Registration:**

Clinical Trials Registry of India, CTRI/2024/08/071848

## Graphical abstract

In this 25‐week, non‐randomized comparative cohort study of 54 adults with MASLD and type 2 diabetes, saroglitazar and dapagliflozin both reduced hepatic steatosis, with no significant between‐group difference in steatosis or liver stiffness. Saroglitazar achieved a significantly greater HbA1c reduction, and no treatment‐emergent adverse events occurred in either arm.

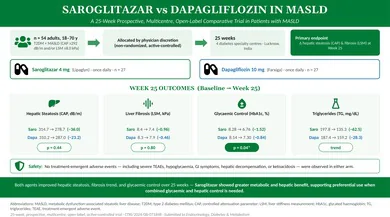

## 1 Introduction

Metabolic dysfunction‐associated steatotic liver disease (MASLD) has become one of the most prevalent causes of chronic liver disease worldwide [1]. It presents as a continuum ranging from non‐progressive steatosis to metabolic dysfunction‐associated steatohepatitis (MASH), with the potential progression to fibrosis, cirrhosis, and hepatocellular carcinoma [2]. The rise in MASLD prevalence is closely associated with the escalating global rates of obesity, type 2 diabetes mellitus (T2DM), and metabolic syndrome. Metabolic dysfunctions significantly contribute to its pathophysiology through mechanisms that include insulin resistance, lipid dysregulation, and inflammatory pathways [3, 4].

India faces a substantial and growing burden of MASLD. Population‐based studies suggest that roughly one‐third of Indian adults (around 36%–38%) have MASLD [5, 6] with regional estimates ranging from 9% to 53% [7]. This is higher than the global average, with a significant proportion demonstrating advanced fibrosis as measured by noninvasive assessments [8]. Urbanization, lifestyle modifications, and genetic predispositions contribute to this heightened incidence. The phenotype of “lean MASLD” in Indian populations, characterized by metabolic irregularities and hepatic steatosis in individuals with normal or low body mass index, complicates diagnosis, and treatment [9].

The clinical implications of MASLD extend beyond liver‐related morbidity and mortality to include heightened cardiovascular risk, complications of type 2 diabetes mellitus, and diminished health‐related quality of life [10]. The psychological impacts, encompassing depression and anxiety associated with chronic disease burden and progression uncertainty, are frequently undervalued yet essential [11].

Current management focuses on lifestyle interventions involving diet, exercise, and weight loss [12]. However, actual compliance with and sustainability of lifestyle modifications remain inconsistent. Pharmacological interventions have consequently garnered attention [13]. Saroglitazar, a dual peroxisome proliferator‐activated receptor alpha/gamma (PPAR‐α/γ) agonist approved in India for the treatment of Diabetes Dyslipidemia, T2DM, MASLD and pre‐cirrhotic MASH, has shown significant effectiveness in improving insulin sensitivity. It reduces hepatic fat accumulation and fibrosis in clinical trials, demonstrating a favorable safety profile [14]. Dapagliflozin and other sodium‐glucose cotransporter‐2 (SGLT2) inhibitors improve glycemic control and reduce liver fat, showing beneficial effects on hepatic steatosis. However, the ability of these agents to improve fibrosis is limited.

This study addressed the paucity of direct comparative data between saroglitazar and dapagliflozin in patients with MASLD and extensive fibrosis. The research aims to provide clinically relevant information to use in therapeutic decision‐making in areas where such information has been absent. Saroglitazar has been shown to have pronounced effects on glycemic control, lipid profile, hepatic enzymes, and fibrosis biomarkers, thereby supporting the use of the drug in the management of complex metabolically impaired hepatic disorders. On the other hand, dapagliflozin improves metabolic and hepatic results, such as steatosis, although its effects on fibrosis are relatively modest.

We hypothesised that, due to its direct hepatic metabolic action via PPAR α/γ activation, saroglitazar would induce more pronounced hepatic steatosis reduction than the indirect hepatic fat benefits of dapagliflozin via glycemic regulation and loss of weight.

## 2 Materials and Methods

### 2.1 Study Design

This 25‐week, multicenter, prospective, open‐label, active‐controlled study was conducted across four diabetes specialty centres in Lucknow, Uttar Pradesh, India. The study evaluated the safety and efficacy of Saroglitazar 4 mg and Dapagliflozin 10 mg, with both medications administered in addition to routine clinical care for diabetes and associated metabolic comorbidities.

### 2.2 Participants

Adults aged 18–70 years were eligible if they had MASLD or nonalcoholic fatty liver disease (NAFLD) confirmed by vibration‐controlled transient elastography (FibroScan). Eligibility required the presence of hepatic steatosis of grade S3 (controlled attenuation parameter [CAP] ≥ 292 dB/m) [15] and/or liver fibrosis of at least stage F2 (liver stiffness measurement [LSM] ≥ 8.0 kPa) [16]. All participants had type 2 diabetes mellitus and were receiving routine clinical care for glycemic management [17]. Patients with decompensated liver disease, significant alcohol consumption, other chronic liver disorders, or unwillingness to provide written informed consent were excluded from the study. Patients were evaluated at baseline and at Week 25 to monitor compliance, efficacy, and safety. Participant screening, allocation, and follow‐up are summarized in the flow diagram (Figure 1), reported in line with the TREND (Transparent Reporting of Evaluations with Nonrandomized Designs) statement for non‐randomized designs.

**FIGURE 1:**
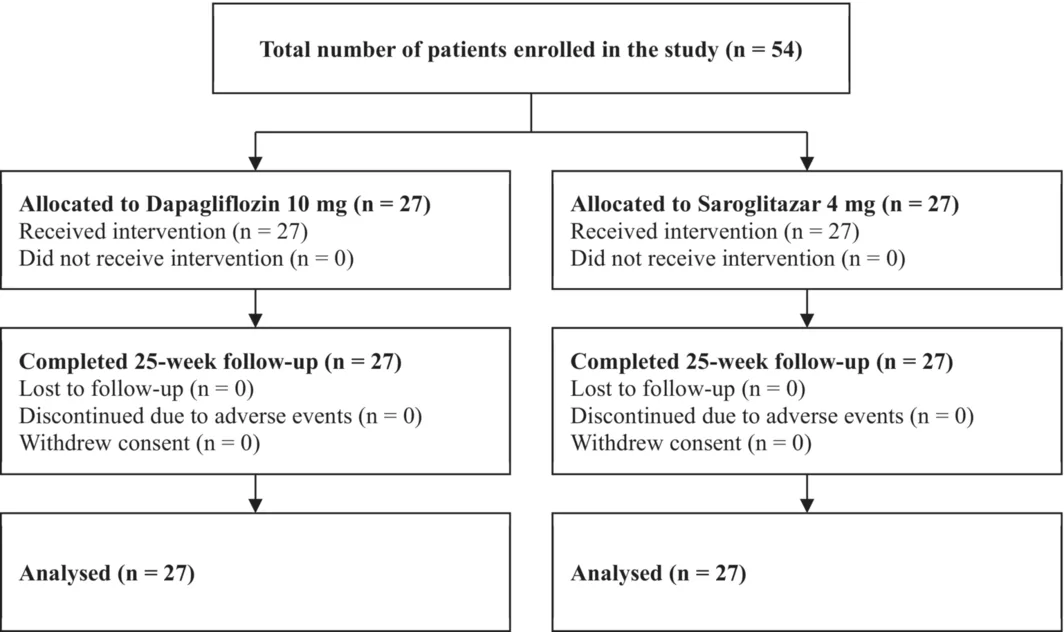
TREND‐aligned flow diagram of the study population.

### 2.3 Intervention and Treatment Protocol

Participants were allocated to receive either Lipaglyn (Saroglitazar 4 mg) or Farxiga (Dapagliflozin 10 mg) once daily for a duration of 25 weeks. Participants were allocated to treatment groups at the physician's discretion (non‐randomized allocation). Compliance and safety were assessed during scheduled appointments throughout the study.

### 2.4 Assessments

At baseline, each participant underwent a thorough assessment comprising medical history, physical examination, anthropometric evaluation, and biochemical investigations including liver function tests, fasting plasma glucose (FPG), and lipid parameters. Liver steatosis and fibrosis were assessed utilizing FibroScan metrics like controlled attenuation parameter (CAP) and liver stiffness measurement (LSM), respectively. Subsequent evaluations were conducted at 12 and 25 weeks to assess biochemical alterations, FibroScan outcomes, and adverse events. Adverse events were assessed through active, structured questioning of participants at each visit, with specific enquiry for edema and fluid retention given saroglitazar's PPAR‐γ component.

### 2.5 Outcomes

The primary endpoint was change from baseline in liver steatosis and fibrosis scores, as measured by FibroScan (CAP and LSM) after 25 weeks of therapy.

Secondary endpoints observed were changes in serum alanine aminotransferase (ALT), aspartate aminotransferase (AST), lipid parameters such as triglycerides (TG) and high‐density lipoprotein cholesterol (HDL‐C), and glycemic parameters such as FPG, glycated haemoglobin (HbA1c), and the incidence of treatment‐emergent adverse events.

### 2.6 Sample Size and Duration

The planned duration of patient follow‐up was 25 weeks, with an overall study duration of 6 months, and 54 participants were enrolled.

### 2.7 Statistical Analysis

Continuous variables are expressed as mean ± standard deviation (SD) and categorical variables as frequencies or percentages. Between‐group comparisons were performed using independent‐samples *t*‐tests or Mann–Whitney *U* tests, as appropriate. Within‐group changes from baseline were evaluated using paired *t*‐tests. Categorical data were analysed with the χ^2^ test or Fisher's exact test. Statistical significance was set at *p* < 0.05. All analyses were performed using SPSS version 25.0.

### 2.8 Ethical Considerations

The study was prospectively registered with the Clinical Trials Registry of India (CTRI/2024/08/071848) and followed the ethical principles of the Declaration of Helsinki, the International Council for Harmonisation (ICH) Good Clinical Practice (GCP) guidelines, and applicable national regulations. The study protocol was reviewed and approved by the Institutional Human Ethics Committees of all participating centres (ECR/1300/Inst/UP/2019). Informed consent was obtained from each participant before the study enrolment.

## 3 Results

The study involved a total of 54 participants, with 27 patients in each of the treatment arms. The gender distribution was comparable between the two groups, with females comprising 59% of the Dapagliflozin cohort and 52% of the Saroglitazar cohort. Baseline ALT was significantly higher in the Saroglitazar group (66.73 ± 47.45 U/L) compared with the Dapagliflozin group (42.85 ± 22.82 U/L), indicating slightly greater hepatocellular injury at baseline. Despite this baseline imbalance, the end‐of‐treatment ALT and AST concentrations were similar in both groups, with the Saroglitazar cohort demonstrating a greater absolute reduction in ALT. There was an overall similarity between the demographic and metabolic characteristics, in terms of age, body mass index (BMI), FPG, and postprandial plasma glucose (PPPG), HbA1c, and lipid parameters as at baseline, as shown in Table 1.

**TABLE 1:** Baseline characteristics of participants by treatment group.

Characteristics	Parameters	Dapagliflozin (mean ± SD)	Saroglitazar (mean ± SD)	*p*
Demographics	Age (years)	53.04 ± 9.92	47.81 ± 11.22	0.076
	Height (cm)	157.14 ± 9.49	159.54 ± 9.43	0.356
	Weight (kg)	73.93 ± 15.02	73.16 ± 13.28	0.842
	BMI (kg/m^2^)	29.86 ± 4.53	28.67 ± 4.19	0.316
	Female, *n* (%)	16 (59%)	14 (52%)	0.580
	Male, *n* (%)	11 (41%)	13 (48%)	
Glycemic parameters	FPG (mg/dL)	147.68 ± 55.07	126.87 ± 33.71	0.100
	PPPG (mg/dL)	197.39 ± 56.45	189.62 ± 76.32	0.672
	HbA1c (%)	8.14 ± 1.65	8.28 ± 2.02	0.781
Hepatic parameters	ALT (U/L)	42.85 ± 22.82	**66.73 ± 47.45** *	0.022
	AST (U/L)	37.58 ± 20.81	50.45 ± 33.16	0.095
Lipid profile	TG (mg/dL)	187.43 ± 65.81	197.83 ± 154.45	0.749
	HDL‐C (mg/dL)	45.43 ± 12.43	50.74 ± 24.68	0.323
FibroScan parameters	CAP (dB/m)	310.22 ± 26.09	314.70 ± 23.11	0.507
	LSM (kPa)	8.31 ± 2.64	8.43 ± 3.25	0.880
FIB‐4 Index		1.5 ± 0.69	1.87 ± 1.23	0.18

*Note:* *p* Values are from independent‐samples *t*‐tests or Mann–Whitney tests as appropriate. Values are presented as mean ± standard deviation unless otherwise indicated. Here, baseline ALT was higher in the Saroglitazar group, indicating more pronounced hepatocellular injury at enrolment. The value in bold indicates significantly higher baseline ALT in the Saroglitazar group than in the Dapagliflozin group (*p* = 0.022).

Abbreviations: ALT, alanine aminotransferase; AST, aspartate aminotransferase; BMI, body mass index; CAP, controlled attenuation parameter; FIB‐4, fibrosis‐4 index; FPG, fasting plasma glucose; HbA1c, glycated haemoglobin; HDL‐C, high‐density lipoprotein cholesterol; LSM, liver stiffness measurement; PPPG, postprandial plasma glucose; TG, triglycerides.

* Statistically significant difference between groups at *p* < 0.05.

### 3.1 Intra‐Group Changes in the Dapagliflozin and Saroglitazar Group

Over 25 weeks of treatment, both dapagliflozin and saroglitazar demonstrated significant intra‐group improvements in hepatic and metabolic parameters. With respect to the primary outcomes, controlled attenuation parameter (CAP) decreased significantly in both treatment arms, indicating a reduction in hepatic steatosis. Liver stiffness measurement (LSM) showed reductions in both treatment groups (−0.46 kPa with dapagliflozin and −0.96 kPa with saroglitazar), though these changes did not reach statistical significance at 25 weeks, thereby indicating limited short‐term antifibrotic effects. Glycemic control improved in both groups, with a statistically significant reduction in HbA1c observed with dapagliflozin and a greater magnitude of reduction with saroglitazar, while changes in fasting and postprandial plasma glucose were modest and did not reach statistical significance. Liver enzymes declined significantly in both treatment arms, reflecting improvement in hepatocellular injury. Triglyceride levels also decreased significantly with both therapies, with a numerically greater reduction observed in the saroglitazar group, whereas changes in HDL‐cholesterol were small and nonsignificant. Detailed quantitative intra‐group changes are presented in Table 2.

**TABLE 2:** Intra‐group comparison of clinical and biochemical parameters from baseline to week 25 in the dapagliflozin and saroglitazar groups.

Category	Parameter	Dapagliflozin				Saroglitazar			
		Baseline (Mean ± SD)	Week 25 (Mean ± SD)	Mean change ± SD	*p*	Baseline (Mean ± SD)	Week 25 (Mean ± SD)	Mean change ± SD	*p*
FibroScan parameters	CAP (dB/m)	310.22 ± 26.09	287.04 ± 39.89	−23.19 ± 32.55	0.001*	314.70 ± 23.11	278.67 ± 38.55	−36.04 ± 41.20	< 0.0001*
	LSM (kPa)	8.31 ± 2.64	7.85 ± 2.73	−0.46 ± 1.46	0.114	8.43 ± 3.25	7.47 ± 1.82	−0.96 ± 3.73	0.384
Anthropometric parameters	BMI (kg/m^2^)	29.87 ± 4.53	29.29 ± 4.84	−0.58 ± 0.84	0.001*	28.66 ± 4.16	28.88 ± 4.17	−0.21 ± 1.33	0.419
Glycemic parameters	HbA1c (%)	8.14 ± 1.65	7.30 ± 0.94	−0.84 ± 1.66	0.0145*	8.28 ± 2.02	6.76 ± 0.96	−1.52 ± 1.80	< 0.0001*
	FPG (mg/dL)	147.68 ± 55.07	126.41 ± 29.89	−21.27 ± 60.49	0.079	126.87 ± 33.70	123.66 ± 26.85	−3.21 ± 35.97	0.647
	PPPG (mg/dL)	197.40 ± 56.45	184.43 ± 51.59	−12.97 ± 70.35	0.347	189.62 ± 76.32	176.82 ± 40.66	−12.81 ± 79.84	0.412
Hepatic parameters	ALT (U/L)	42.85 ± 22.82	34.19 ± 17.27	−8.65 ± 21.27	0.044*	66.73 ± 47.45	36.22 ± 21.83	−30.51 ± 42.37	0.001*
	AST (U/L)	37.58 ± 20.81	28.30 ± 11.14	−9.28 ± 16.68	0.0077*	50.45 ± 33.16	30.29 ± 12.40	−20.16 ± 30.41	0.002*
Lipid profile	TG (mg/dL)	187.43 ± 65.81	159.17 ± 75.10	−28.27 ± 59.02	0.022*	197.83 ± 154.45	135.34 ± 61.15	−62.48 ± 155.80	0.047*
	HDL‐C (mg/dL)	45.43 ± 12.43	42.96 ± 7.45	−2.47 ± 13.42	0.348	50.74 ± 24.68	47.38 ± 12.59	−3.36 ± 25.82	0.505
FIB‐4 index		1.5 ± 0.69	1.35 ± 0.52	−0.15 ± 0.61	0.37	1.87 ± 1.23	1.61 ± 1.5	−0.26 ± 1.4	0.49

Abbreviations: ALT, alanine aminotransferase; AST, aspartate aminotransferase; BMI, body mass index; CAP, controlled attenuation parameter; FIB‐4, fibrosis‐4 index; FPG, fasting plasma glucose; HbA1c, glycated haemoglobin; HDL‐C, high‐density lipoprotein cholesterol; LSM, liver stiffness measurement; PPPG, postprandial plasma glucose; TG, triglycerides.

* Statistically significant difference between groups at *p* < 0.05.

At Week 25, the between‐group comparisons are summarized in Table 3a. For the primary outcomes, reductions in CAP and LSM were greater in the saroglitazar group, although these differences did not reach statistical significance. Among the secondary outcomes, HbA1c levels were significantly lower with saroglitazar compared with dapagliflozin (6.76% ± 0.96% vs. 7.30% ± 0.94%, *p* = 0.04), highlighting its stronger effect on glycemic control. Despite higher baseline ALT values in the saroglitazar group, both treatments led to similar normalization of ALT and AST by Week 25, indicating comparable hepatic improvement.

**TABLE 3a:** Inter‐group comparison of clinical and biochemical parameters at week 25.

Parameters	Dapagliflozin	Saroglitazar	*p*
	Mean ± SD	Mean ± SD	
Demographic parameters			
BMI (kg/m^2^)	29.29 ± 4.84	28.88 ± 4.18	0.74
Glycemic parameters			
FPG (mg/dL)	126.41 ± 29.89	123.66 ± 26.85	0.72
PPPG (mg/dL)	184.43 ± 51.59	176.82 ± 40.66	0.55
HbA1c (%)	7.30 ± 0.94	6.76 ± 0.96	0.04*
Liver parameters			
ALT (U/L)	34.19 ± 17.27	36.22 ± 21.83	0.71
AST (U/L)	28.30 ± 11.14	30.29 ± 12.40	0.54
Lipid parameters			
TG (mg/dL)	159.17 ± 75.10	135.34 ± 61.15	0.21
HDL‐C (mg/dL)	42.96 ± 7.45	47.38 ± 12.59	0.12
Fibroscan			
LSM (kPa)	7.85 ± 2.73	7.47 ± 1.82	0.80
CAP (dB/m)	287.04 ± 39.89	278.67 ± 38.55	0.44
FIB‐4 Index	1.35 ± 0.52	1.61 ± 1.5	0.398

*Note:* Data are presented as mean ± SD. Between‐group comparisons at Week 25 were made using the independent‐samples *t*‐test.*p < 0.05, statistically significant difference between the Dapagliflozin and Saroglitazar groups.

Abbreviations: ALT, alanine aminotransferase; AST, aspartate aminotransferase; BMI, body mass index; CAP, controlled attenuation parameter; FIB‐4, fibrosis‐4 index; FPG, fasting plasma glucose; HbA1c, glycated haemoglobin; HDL‐C, high‐density lipoprotein cholesterol; LSM, liver stiffness measurement; PPPG, postprandial plasma glucose; SD, standard deviation; TG, triglycerides.

Lipid parameters showed a trend toward greater TG reduction with saroglitazar, although the difference was not statistically significant. Other metabolic and hematologic measures such as FPG and PPPG, BMI, and FIB‐4 index were also similar between the two groups.

No complications such as hypoglycemia, gastrointestinal symptoms, hepatic decompensation, ketoacidosis, edema, or any treatment‐related adverse events were reported in either treatment arm on active questioning at each visit.

## 4 Discussion

This 25‐week, multicenter, active‐controlled study compared the metabolic and hepatic effects of saroglitazar and dapagliflozin in patients with MASLD and demonstrated clinically meaningful improvements in steatosis, glycemic parameters, liver enzymes, and lipid metabolism with both therapies. Saroglitazar showed a numerically greater within‐group reduction in hepatic fat (CAP) than dapagliflozin (36.04 vs. 23.19 dB/m) and a significantly greater reduction in HbA1c (−1.52% vs. −0.84%, *p* = 0.04); however, the between‐group difference in CAP reduction did not reach statistical significance (*p* = 0.44), so superiority of either agent for hepatic steatosis reduction cannot be concluded from this study. Both agents led to similar reductions in transaminases as well as comparable, nonsignificant reductions in fibrosis indices (LSM, *p* = 0.80). These results highlight unique similarities in action, different metabolic patterns, and the future potential for selective pharmacologic usage of each drug in the treatment of MASLD.

The achieved decreases in CAP and HbA1c with the use of saroglitazar are biologically feasible and can be explained by the sensitivity of this agonist to two peroxisome proliferator‐activated receptors, alpha and gamma. PPAR‐α activation promotes hepatic beta‐oxidation, inhibits de novo lipogenesis, and enhances the breakdown of TG, hence directly treating hepatic steatosis. Co‐stimulation of PPAR‐γ results in improved peripheral insulin sensitivity, adiponectin secretion, and reduced hepatic lipotoxicity, and these effects promote hepatic lipid depletion. These interactive processes transform a synergistic metabolic environment that can reduce TG load on the liver and at the same time enhance glycemic control [18, 19]. In contrast, dapagliflozin exerts its hepatic benefits indirectly through its renal mechanism of action. Dapagliflozin (inhibition of sodium‐glucose cotransporter 2, SGLT2) enhances urinary glucose excretion, creates a sustained caloric deficit of about 200–300 kcal per day, and promotes a metabolic shift toward the use of lipids and ketones [20, 21]. This introduced metabolic pattern that imitates fasting promotes insulin sensitivity in the body and moderates adiposity in the liver. These mechanistic actions plausibly explain the numerically greater within‐group changes in CAP and TG observed with saroglitazar in this study, although the between‐group difference for CAP did not reach statistical significance; dapagliflozin's hepatic effect was of relatively smaller magnitude and secondary to weight‐related changes in metabolism [22].

The hepatic steatosis results in the present study align well with published literature. Gawrieh et al. [23] reported a CAP reduction of −19.7 dB/m with saroglitazar over 24 weeks in NAFLD patients, which is comparable to our more substantial improvement (−36.04 dB/m) in a population with severe steatosis at baseline. Real‐world data from Goyal et al. [24] also showed consistent reductions in hepatic fat with saroglitazar. For dapagliflozin, the CAP reduction in our study (−23.19 dB/m) exceeds the pooled effect of −13.77 dB/m reported in the meta‐analysis by Ong Lopez et al. [25], possibly due to higher baseline hepatic fat and longer therapy duration in our cohort. Both findings are consistent with the systematic review by Zhou et al. [26], which highlighted that SGLT2 inhibitors demonstrate greater improvements in steatosis when baseline fat burden is severe, and treatment extends beyond 24 weeks.

Fibrosis outcomes in the present study showed reductions in LSM in both groups (−0.46 kPa with dapagliflozin and −0.96 kPa with saroglitazar), indicating favourable trends toward fibrosis improvement. Although these changes did not achieve statistical significance, such findings are expected, given that substantial fibrosis regression typically requires ≥ 52 weeks of therapy in most pharmacologic trials. Our findings are consistent with the meta‐analysis by Jin et al. [27], which showed that SGLT2 inhibitors exert modest, dose‐ and duration‐dependent antifibrotic effects, particularly in early‐stage fibrosis. Similarly, the saroglitazar RCT by Gawrieh et al. reported small but favourable LSM trends over 24 weeks [23]. Shimizu et al. similarly reported that dapagliflozin significantly reduced liver stiffness on transient elastography over 24 weeks in patients with type 2 diabetes and nonalcoholic fatty liver disease, supporting a modest but consistent antifibrotic signal for SGLT2 inhibitors [28].

With respect to glycemic control, both drugs significantly reduced HbA1c, but the effect was notably greater with saroglitazar (−1.52% vs. −0.84%). The magnitude of HbA1c reduction reported in our study aligns well with existing evidence from both randomized controlled trials and real‐world data on saroglitazar. In a randomized controlled trial (RCT) by Gawrieh et al., saroglitazar demonstrated an HbA1c reduction close to −1.8%, consistent with significant glycemic improvement [23]. The dapagliflozin‐related HbA1c reduction in our study also matches prior evidence by Das et al. [29], who observed a reduction of −0.74%, and multiple RCTs in T2DM patients consistently demonstrate improvements of −0.6% to −1.0%. These findings reinforce the differential metabolic potency of the two agents, with saroglitazar offering a direct insulin‐sensitizing effect, while dapagliflozin improves glucose disposal through glucosuria and negative energy balance.

Body weight and BMI changes further reflect mechanistic differences. Dapagliflozin produced a modest BMI reduction in our study, consistent with its known 2–3 kg weight‐lowering effect reported in SGLT2 inhibitor trials. Saroglitazar, in contrast, is characteristically weight‐neutral, as confirmed in our population. This distinction is clinically meaningful when selecting therapy for overweight or obese MASLD patients.

Both drugs demonstrated strong hepatoprotective effects, with clinically relevant reductions in ALT and AST in both treatment arms. Saroglitazar produced a larger absolute decline in liver enzymes (ALT −30.51 U/L; AST −20.16 U/L), consistent with prior studies such as Goyal et al. (ALT −45%, AST −58%) and the saroglitazar phase 2 RCT [24, 30]. Dapagliflozin also significantly improved liver enzymes (ALT −8.65 U/L; AST −9.28 U/L), consistent with findings from Das et al. and SGLT2 inhibitor meta‐analyses showing reductions in ALT driven by decreased hepatic fat and inflammation [29]. These consistent enzyme declines confirm that both classes confer hepatocellular protection, albeit through different pathways. The modest, non‐significant change in FIB‐4 index in both groups (Table 2) is consistent with this transaminase pattern. We found that baseline and/or Week‐25 platelet values for 5 participants (4 Dapagliflozin, 1 Saroglitazar) were not physiologically plausible; after excluding these participants, FIB‐4 in both groups showed a small, nonsignificant decline (Table 3b) consistent with the concurrent AST/ALT trend, with no evidence of an independent platelet‐driven effect.

**TABLE 3b:** ANCOVA‐adjusted between‐group comparison at week 25 (adjusting for baseline value).

Parameter	Unadjusted diff (Saro−Dapa)	Unadj. *p*	ANCOVA‐adjusted diff (95% CI)^b^	Adj. *p*
BMI (kg/m^2^)	−0.41	0.740	+0.80 [0.18, 1.42]	0.0125*
FPG (mg/dL)	−2.75	0.723	−0.68 [−16.57, 15.20]	0.9317
PPBG (mg/dL)	−7.61	0.550	−6.74 [−32.08, 18.59]	0.5954
HbA1c (%)	−0.54	0.051	−0.55 [−1.05, −0.05]	0.0325*
ALT (U/L)	+2.02	0.707	−3.57 [−13.81, 6.67]	0.4868
AST (U/L)	+1.99	0.537	−0.56 [−6.47, 5.36]	0.8505
TG (mg/dL)	−23.43	0.209	−25.61 [−61.31, 10.08]	0.1558
HDL‐C (mg/dL)	+4.42	0.124	+3.96 [−1.72, 9.65]	0.1678
LSM (kPa)	−0.38	0.549	−0.45 [−1.35, 0.45]	0.3220
CAP (dB/m)	−8.37	0.437	−11.20 [−31.15, 8.74]	0.2648
FIB‐4 Index^a^	−0.06	0.749	−0.15 [−0.43, 0.14]	0.3069

*Note:* Asterisk (*) denotes a statistically significant difference between groups at *p* < 0.05. Model: Week‐25 value ≈ baseline value + treatment group (ordinary least squares ANCOVA). Positive difference = higher in Saroglitazar than Dapagliflozin at Week 25, after adjusting for each parameter's own baseline value.

Abbreviations: ALT, alanine aminotransferase; AST, aspartate aminotransferase; BMI, body mass index; CAP, controlled attenuation parameter; FIB‐4, fibrosis‐4 index; FPG, fasting plasma glucose; HbA1c, glycated haemoglobin; HDL‐C, high‐density lipoprotein cholesterol; LSM, liver stiffness measurement; PPPG, postprandial plasma glucose; TG, triglycerides.

^a^ FIB‐4 computed on *n* = 49 (23 Dapagliflozin, 26 Saroglitazar) after excluding 5 participants (4 Dapagliflozin, 1 Saroglitazar) whose recorded baseline and/or Week‐25 platelet values were not physiologically plausible.

^b^ Between‐group difference (Saroglitazar − Dapagliflozin) in adjusted mean Week‐25 values, with 95% confidence interval, estimated by analysis of covariance (ANCOVA) with treatment group as the fixed factor and the respective baseline value as the covariate.

* *p* < 0.05.

An important methodological consideration in our study was the significant baseline imbalance in ALT, which was higher in the saroglitazar group (66.73 U/L vs. 42.85 U/L; *p* = 0.022), suggesting greater hepatocellular injury at enrolment. Despite this disadvantage, saroglitazar achieved enzyme normalization comparable to dapagliflozin (36.22 vs. 34.19 U/L at week 25), implying potent hepatic benefit even in those with more severe biochemical injury. This imbalance likely reflects real‐world prescribing behaviour, where clinicians preferentially select saroglitazar for patients with more pronounced metabolic derangements. Although this enhances external validity, it limits the internal validity of direct intergroup comparisons.

The lipid profile changes in our study strengthen the therapeutic differentiation between these agents. Saroglitazar produced a substantial reduction in TG (−62.48 mg/dL), consistent with its PPAR‐α–mediated enhancement of fatty acid oxidation, and comparable to the 50–70 mg/dL reductions reported in prior saroglitazar studies. Dapagliflozin reduced TG modestly (−28.27 mg/dL), which aligns with meta‐analytic estimates of −10 to −20 mg/dL for SGLT2 inhibitors [31]. HDL changes were mild and non‐significant in both groups, consistent with published literature that reports minimal HDL modulation in short‐duration studies.

Saroglitazar's PPAR‐γ component raises a theoretical risk of the pedal edema and fluid retention seen with conventional PPAR‐γ agonists; no edema was observed in either arm in this study on active questioning at each visit, consistent with prior evidence that saroglitazar, unlike conventional PPAR‐γ agonists, is largely devoid of pedal edema and weight gain at approved doses [32].

Clinically, these findings support a personalized approach to MASLD treatment based on individual comorbidities. Saroglitazar may be preferred for patients needing significant glycemic control (especially HbA1c > 8%), those with hypertriglyceridemia, or high liver fat, due to its direct liver‐targeted metabolic effects and weight‐neutral profile, making it suitable for lean or normal‐weight patients. Dapagliflozin could be more beneficial for overweight or obese patients, or those with cardiovascular, kidney, or heart failure comorbidities, given its proven cardiorenal benefits and modest weight loss. Integrating metabolic, liver, and systemic risk factors is essential to selecting the best treatment for each MASLD patient.

This study offers a valuable direct, real‐world comparison of saroglitazar and dapagliflozin, addressing an important gap in MASLD treatment evidence. By selecting patients with significant steatosis and moderate fibrosis, it focused on a clinically relevant group. Use of validated tools like FibroScan and detailed biochemical analysis enhances result accuracy. The multicentre design improves generalizability, while combined evaluation of metabolic, hepatic, and anthropometric outcomes provides a comprehensive picture. Conducted in routine clinical settings, findings are highly applicable to everyday practice, supporting evidence‐based decision‐making.

This study has some limitations to consider. Being a non‐randomized, open‐label design, it is vulnerable to selection and observational biases. Differences at baseline, such as higher ALT levels in the saroglitazar group, suggest underlying clinical variability. Because only 54 participants were ultimately enrolled, the study was not statistically powered, thereby limiting the precision of estimates and the ability to detect smaller between‐group effect sizes. The 25‐week duration was suitable for observing short‐term biochemical changes but may be too brief to assess long‐term disease effects, and no histological confirmation was possible. Serum adiponectin was not measured in this study, so the extent to which saroglitazar's glycemic benefit is mediated by adiponectin induction could not be assessed and it is recommended to be measured in future comparative studies. Post hoc correlation analysis found only a weak, nonsignificant association between change in CAP and change in HbA1c in either group (Dapagliflozin *r* = 0.09, *p* = 0.66; Saroglitazar *r* = 0.22, *p* = 0.28). A similarly weak association was seen between change in CAP and change in triglycerides in the Dapagliflozin group (*r* = 0.02, *p* = 0.93), while a borderline positive correlation was observed in the Saroglitazar group (*r* = 0.38, *p* = 0.054); this is consistent with, but not conclusive evidence for, a contribution of saroglitazar's triglyceride‐lowering action to its hepatic fat reduction. ANCOVA adjusting for each parameter's own baseline value (Table 3b) confirmed that the between‐group difference in HbA1c reduction remained statistically significant after adjustment (adjusted difference −0.55%, 95% CI −1.05 to −0.05, *p* = 0.033), addressing the baseline imbalance, while adjusted differences for ALT, AST, CAP, and LSM remained non‐significant. This adjusted analysis also revealed a significant between‐group difference in Week‐25 BMI (adjusted difference + 0.80 kg/m^2^, *p* = 0.0125, higher in Saroglitazar), consistent with dapagliflozin's known weight‐lowering mechanism. Use of surrogate endpoints and data from a single geographic location further restricts how broadly the results can be applied. Despite these issues, this study provides valuable early insights and highlights the need for larger, longer, randomized trials to better evaluate treatment efficacy and safety.

These results nonetheless offer practical guidance for personalizing therapy in MASLD. Saroglitazar may be preferred for patients requiring intensive glycemic control (e.g., HbA1c > 8%), hypertriglyceridemia or direct hepatic metabolic modulation because its dual PPAR‐α/γ activity improves both insulin resistance and lipid metabolism without promoting weight gain or weight loss. Dapagliflozin remains valuable for overweight or obese patients or those with cardiovascular disease, chronic kidney disease or heart failure, owing to its weight‐lowering effect and established cardiorenal benefits. Overall, a comorbidity‐driven approach that integrates hepatic and systemic metabolic priorities is warranted. Longer, randomized, histology‐based studies are needed to confirm these comparative benefits and to determine whether combination or sequential therapy yields additional benefit in MASLD management.

## 5 Conclusion

Both treatments led to clear improvements in blood sugar control and liver health among patients with MASLD. Saroglitazar was associated with a significantly greater reduction in HbA1c, indicating a possible glycemic advantage. Hepatic steatosis (CAP) and fibrosis (LSM) improved with both agents, with numerically greater within‐group reductions seen with saroglitazar; however, the between‐group differences for these hepatic parameters were not statistically significant, so superior hepatic efficacy cannot be claimed for either agent. Larger, randomized, adequately powered trials are needed to confirm whether either agent confers a true hepatic advantage.

## Author Contributions

**Mukulesh Gupta:** conceptualization, investigation, funding acquisition, writing – original draft, methodology, validation, visualization, writing – review and editing, software, formal analysis, project administration, data curation, supervision, resources. **Kumar Prafull Chandra:** methodology, data curation, investigation, formal analysis, funding acquisition, writing – original draft, writing – review and editing. **Rajiv Awasthi:** conceptualization, methodology, data curation, investigation, formal analysis, supervision, visualization. **Arunkumar R. Pande:** software, validation, writing – original draft, writing – review and editing. **Nitin Ranjan Gupta:** funding acquisition, writing – original draft, writing – review and editing. **Vivek Agarwal:** software, validation, funding acquisition, writing – original draft, writing – review and editing. **Dinesh Kumar:** conceptualization, methodology, data curation, investigation, formal analysis, project administration, resources, writing – original draft, writing – review and editing.

## Funding

The authors have nothing to report.

## Disclosure

Artificial Intelligence‐Generated Content Disclosure: No AI‐generated content was used in the preparation of this manuscript beyond standard spelling/grammar checking.

## Ethics Statement

This study was prospectively registered with the Clinical Trials Registry of India (CTRI/2024/08/071848) and was conducted in accordance with the ethical principles of the Declaration of Helsinki and the International Council for Harmonisation (ICH) Good Clinical Practice (GCP) guidelines. The study protocol was reviewed and approved by the Institutional Human Ethics Committees of all participating centres (ECR/1300/Inst/UP/2019). Written informed consent was obtained from all participants prior to enrolment.

## Conflicts of Interest

The authors declare no conflicts of interest.

## Data Availability

The data that support the findings of this study are available on request from the corresponding author. The data are not publicly available due to privacy or ethical restrictions.
